# Relationship between sleep duration and dietary intake in 4- to 14-year-old Danish children

**DOI:** 10.1017/jns.2013.23

**Published:** 2013-12-06

**Authors:** Camilla Hoppe, Berit W. Rothausen, Anja Biltoft-Jensen, Jeppe Matthiessen, Margit V. Groth, Jean-Philippe Chaput, Inge Tetens

**Affiliations:** 1 Division of Nutrition, National Food Institute, Technical University of Denmark, Søborg, Denmark; 2 Healthy Active Living and Obesity Research Group, Children's Hospital of Eastern Ontario Research Institute, Ottawa, ON, Canada

**Keywords:** Diet, Obesity, Sleep, Children, Adolescents, Denmark

## Abstract

A negative association between sleep duration and BMI has been observed in children. However, knowledge about the association between sleep duration and diet is limited. The objective was to examine the association between sleep duration and intake of foods and nutrients in children. In the present cross-sectional study, dietary intake and sleep duration were recorded by the parents for seven consecutive days in a food and sleep record in a representative sample of 802 4- to 14-year-old children. No sex differences were found regarding age and sleep duration. Sleep duration was negatively correlated to age (ρ = –0·68; *P* < 0·001) and BMI (ρ = –0·41; *P* < 0·001). In multiple linear regression analyses, sleep duration was not associated with energy intake (b = –0·015; *P* = 0·20), but there was a trend towards a positive association with intake of dietary fibre (b = 0·006; *P* = 0·05) and vegetables (b = 0·011; *P* = 0·05), and a negative association with intake of poultry (b = –0·002; *P* = 0·02), and a trend towards a negative association with intake of liquid ‘discretionary calories’ (b = –0·01; *P* = 0·05). Furthermore, in a comparison of dietary intake between age-dependent tertiles of sleep duration, only intake of liquid ‘discretionary calories’ was significantly lower in long sleepers than in short and medium sleepers (*P =* 0·03). In conclusion, sleep duration was not associated with energy intake and the proposal that children with short sleep duration have less healthy eating habits than children with longer sleep duration was only weakly supported by the present findings.

The prevalence of childhood obesity is emerging as a major health problem^(^^1^^)^. In several studies, a negative or U-shaped relationship between sleep duration and weight status has been observed in infants^(^^2^^)^, children^(^^3^^,^^4^^)^, adolescents^(^^4^^,^^5^^)^ and adults^(^^3^^,^^6^^)^. Moreover, meta-analyses analysing data from studies in children have concluded that shorter sleep duration was associated with higher odds of being obese^(^^3^^)^. This might have long-term implications^(^^7^^)^ because tracking of overweight from childhood to adulthood occurs^(^^8^^)^, thereby increasing the risk of developing certain non-communicable diseases, as overweight is associated with certain non-communicable diseases^(^^9^^)^. The potential influence of shorter sleep duration on body weight balance is not fully understood. One possible underlying mechanisms is that shorter sleep duration makes an impact on the hormonal regulation of appetite. Indeed, lack of sleep has been reported to decrease leptin levels, increase ghrelin levels, alter glucose homeostasis, and activate the orexin system^(^^10^^)^. Furthermore, shorter sleep duration might also promote overeating and weight gain by increasing the time available for eating, especially in the evening where sedentary activities, such as watching television, and snacking on highly palatable and energy-dense foods are common^(^^11^^)^.

When shortened sleep duration leads to sleepiness and/or fatigue during daytime this may limit the motivation for being physically active and promote sedentary behaviours instead. It has therefore also been speculated that there is an association between sleep duration, BMI and physical activity; however, results from studies concerning this have been contradictory^(^^5^^,^^12^^)^. Only a few studies have examined the association between sleep duration and dietary quality^(^^13^^–^^15^^)^. This issue is particularly relevant from a public health standpoint because increased energy intake appears to be the most plausible explanation as to why children with shorter sleep duration have a higher risk of becoming obese.

The main aim of the present study was to examine the relationship between intake of different foods and nutrients, and sleep duration in Danish children. We hypothesised that short sleepers have higher energy intake and less healthy food intake compared with long sleepers.

## Materials and methods

### Sample

The present study was based on data from the Danish National Survey of Dietary Habits and Physical Activity (DANSDA) 2003–2008, which is a nationwide and representative cross-sectional survey. The data collection was evenly distributed during the period. The study population comprised a simple random sample of 4- to 14-year-old children retrieved from The Central Office of Civil Registration. In comparison with census data from Statistics Denmark, the distribution of sex and age of the participants could be characterised as representative and parental education as close to representative for the Danish population of children aged 4–14 years. Data from a total of 802 children were available and included in the present study.

The study was conducted according to the guidelines laid down in the Declaration of Helsinki and was approved by the Danish Data Protection Agency. The Danish National Committee on Health Research Ethics decided that DANSDA did not require their approval.

### Anthropometric variables

Information about height and weight was obtained through a personal face-to-face interview with one of the parents. The prevalence of overweight and obesity in the study population was defined according to international age- and sex-specific BMI cut-off values^(^^16^^)^, corresponding to BMI values of 25 and 30 kg/m^2^, respectively, for adults aged ≥18 years.

### Sleep duration

Sleep duration was reported in hours and minutes in a diary integrated in a food record. Every day, on the same 7 d as the food record was completed, a simple question about sleep duration during the last night and day was answered: ‘what length of time did you sleep for during the last 24 h (please include daytime napping)?’. Children and their parents were instructed in person by trained interviewers on how to complete food and sleep recordings. The parents were responsible for completing the records and for deciding to what extent their children were capable of assisting. Thus, parents reported all or the majority of the dietary intake and sleep for the youngest children, but somewhat less for the older children. Mean sleep duration per d was calculated and used for subsequent analyses. Days where children were in bed with illness were omitted. For each child, a minimum of 4 d with measurements of diet and sleep duration was required for inclusion in the statistical analyses. Children with short, medium and long sleep duration were defined within each year of age according to the tertiles of sleep duration.

### Dietary intake

Dietary intake was recorded every day for seven consecutive days in food records with pre-coded response categories, which included open-answer options. For food items not included in the pre-coded food record, the participants wrote the type of food and portion size eaten in open-answer categories. The amount of foods eaten was given in predefined household measures (cups, spoons, slices, etc.) or estimated from photographs of various portion sizes. Children also received a food record booklet to take to school or to other places outside of the home on the days of assessment. Details about the method and calculation of intake of food and nutrients are described elsewhere^(^^17^^)^. In order to characterise the children's dietary intake, energy intake, macronutrients and major food groups as well as energy-dense foods were included in the analyses.

### Parental education

Parental educational level was recorded as the educational level of parent in the household with the highest education, and divided into four levels: (1) basic school (10 years or less of total education); (2) vocational education, upper secondary school (10–12 years); (3) short higher education (13–15 years) (primarily theoretical); and (4) long higher education (15+ years) (primarily theoretical).

### Statistical analysis

All data were analysed with SPSS (version 20; IBM SPSS Statistics, Inc.) and a significance level of *P* < 0·05 was used (two-tailed tests). Normality was checked visually with histograms as well as with Kolgomorov–Smirnoff tests. As commonly found, the data for most foods and nutrients were not normally distributed (positively skewed). Logarithmic and square root transformations were attempted, but did not produce distributions with sufficient normality. For this reason and insufficient homogeneity of variance, non-parametric analyses were performed. BMI *Z*-scores were calculated. Spearman's ρ correlation coefficients were calculated to analyse associations between sleep duration and BMI and between sleep duration and age. The χ^2^ test was used to analyse differences between weight status, parental education and sleep duration and sex. The Kruskal–Wallis test was used to analyse differences in BMI between parental education. Multiple linear regression analyses were performed with Bonferroni adjustment for multiple tests separately for each dietary variable with sleep duration (h/d) as the dependent variable and with BMI (kg/m^2^), age (years), sex (male, female), energy intake (kJ/d) and parental education level (1, 2, 3, 4; see Parental education section above) as forced covariates in one model, and with age (years), sex (male, female), energy intake (kJ/d) and parental education level (1, 2, 3, 4) as forced covariates in another model. Normality of the residuals from the models was regarded as sufficient. In order to further elucidate the dietary intakes in relation to sleep duration, the Kruskal–Wallis test was applied to test for statistically significant differences in dietary intake between age-dependent tertiles of sleep duration. Results are provided as mean values and standard deviations.

## Results

### Descriptive characteristics

Characteristics of the study population are given in Table 1, and are shown for the three age groups: 4–6 years, 7–10 years and 11–14 years. This is the standard age grouping of children in the National Survey of Dietary Habits and Physical Activity, since these age groups correspond to the Danish institutions for children: kindergarten (4–6 years), introductory school period (7–10 years) and intermediate school period (11–14 years). Within each age group, no differences between boys and girls were found regarding age (*P ≥* 0·29), height (*P ≥* 0·52), weight (*P ≥* 0·30), BMI (*P ≥* 0·17), parental education (*P ≥* 0·39) and sleep duration (*P ≥* 0·95). Therefore, further analyses were conducted with both sexes combined.

**Table 1.** Characteristics of the study population (Mean values and standard deviations; number of subjects and percentages)

Age group…	4–6 years	7–10 years	11–14 years
Subjects (*n*)	212	289	301
Sex (%)			
Male	50	54	45
Female	50	46	55
Height (cm)			
Mean	118·6	139·4	161·4
sd	8·9	9·3	9·7
Weight (kg)			
Mean	22·1	33·0	50·5
sd	4·1	7·4	10·9
BMI (kg/m^2^)			
Mean	15·6	16·8	19·3
sd	1·9	2·6	3·1
Weight status*			
Overweight (%)			
Male	7·5	12·9	19·4
Female	16·0	14·9	15·0
Obesity (%)			
Male	0·0	3·8	2·2
Female	3·8	4·5	4·2
Sleep duration (h)			
Mean	10·2	9·6	8·9
sd	0·6	0·5	0·7
Parental education (%)†			
(1) Basic school	9	9	11
(2) Vocational education	41	43	42
(3) Short higher education	8	10	15
(4) Long higher education	42	38	32

* Weight status according to international cut-off values^(^^16^^)^.

† Parental educational level (*n* 731): (1) basic school (10 years or less of total education); (2) vocational education, upper secondary school (10–12 years); (3) short higher education (13–15 years) (primarily theoretical); (4) long higher education (15+ years) (primarily theoretical).

### Prevalence of overweight and obesity

Prevalence of overweight and obesity is given in Table 1. No sex differences were observed with regard to weight status for the 7- to 10-year-old and 11- to 14-year-old children (*P* = 0·59 and *P* = 0·93), but there were more overweight and obese girls than boys in the 4- to 6-year-old children (*P* = 0·004).

### Sleep duration, age and BMI

Age was negatively correlated to sleep duration (ρ = –0·68; *P* < 0·001). An inverse relationship between sleep duration and BMI *Z*-scores (4–14 years: ρ = –0·41, *P* < 0·001; 4–6 years: ρ = –0·19, *P =* 0·79; 7–10 years: ρ = –0·16, *P =* 0·008; 11–14 years: ρ = –0·14, *P =* 0·013) was also observed, but was not statistically significant for the youngest age group. Furthermore, length of parental education was negatively correlated with BMI of the child (*P* < 0·05). However, there were no differences between short, medium and long sleepers regarding parental education (*P* = 0·44).

### Sleep duration and dietary intake

Multiple linear regression analyses were performed with Bonferroni adjustment for multiple tests separately for each dietary variable with sleep duration (h/d) as the dependent variable and with BMI (kg/m^2^), age (years), sex (male, female), energy intake (kJ/d) and parental education (1, 2, 3, 4) as forced covariates in model A, and with age (years), sex (male, female), energy intake (kJ/d) and parental education (1, 2, 3, 4) as forced covariates in model B. Sleep duration was not associated with energy intake (model A: b = –0·015, *P* = 0·20; model B: b = –0·015, *P* = 0·20), but there was a trend towards a positive association with intake of dietary fibre (model A: b = 0·006, *P* = 0·05; model B: b = 0·006, *P* = 0·05) and vegetables (model A: b = 0·011, *P* = 0·05; model B: b = 0·009, *P* = 0·05), and a negative association with intake of poultry (model A: b = –0·002, *P* = 0·02; model B: b = –0·002, *P* = 0·02) and a trend towards a negative association with liquid ‘discretionary calories’ (model A: b = –0·010, *P* = 0·05; model B: b = –0·011, *P* = 0·06), as shown in Table 2. Excluding BMI as covariate did not change the results markedly.

**Table 2.** Coefficients from multiple linear regression analyses performed separately for each dietary variable with sleep duration (h/d) as the dependent variable and with BMI (kg/m^2^), age (years), sex (male, female), energy intake (kJ/d) and parental education (1, 2, 3, 4)* as forced covariates in model A, and with age (years), sex (male, female), energy intake (kJ/d) and parental education (1, 2, 3, 4)* as forced covariates in model B (*n* 802)

	Model A		Model B	
	b	*P*†	b	*P*†
Energy (MJ/d)	−0·015	0·20	−0·015	0·20
Protein (E%)‡	0·006	0·53	−0·007	0·56
Fat (E%)‡	−0·001	0·16	−0·008	0·99
SFA (E%)‡	−0·002	0·82	−0·001	0·92
MUFA (E%)‡	−0·009	0·47	−0·007	0·55
PUFA (E%)‡	−0·002	0·90	−0·008	0·99
Carbohydrate (E%)‡	0·002	0·63	0·002	0·72
Added sugars (E%)‡	−0·003	0·53	−0·003	0·55
Dietary fibre (g per10 MJ)	0·006	0·05	0·006	0·05
High-fat milk and milk products (g per10 MJ)	<0·001	0·47	<0·001	0·40
Low-fat milk and milk products (g per10 MJ)	−0·008	0·31	−0·008	0·27
Rye bread (g per10 MJ)	0·001	0·14	0·001	0·16
Wheat bread (g per10 MJ)	<0·001	0·99	−0·003	0·95
Sugary breakfast cereals (g per10 MJ)§	0·001	0·98	0·001	0·99
Fruit (g per10 MJ)	−0·006	0·68	−0·007	0·65
Vegetables (g per10 MJ)	0·011	0·05	0·009	0·05
Meat and meat products (g per10 MJ)	0·007	0·90	0·004	0·94
Poultry (g per10 MJ)	−0·002	0·02	−0·002	0·02
Fish and seafood (g per10 MJ)	0·002	0·14	0·002	0·13
Fast food and light meals (g per10 MJ)∥	0·009	0·71	0·010	0·71
Snacks (g per10 MJ)¶	−0·001	0·49	−0·001	0·54
Sweets and chocolate (g per10 MJ)	0·001	0·46	0·001	0·47
Solid ‘discretionary calories’ (g per 10 MJ)**	<0·001	0·62	<0·001	0·66
Liquid ‘discretionary calories’ (g per 10 MJ)††	−0·010	0·05	−0·011	0·06

E%, energy percentage.

* Parental educational level: (1) basic school (10 years or less of total education); (2) vocational education, upper secondary school (10–12 years); (3) short higher education (13–15 years) (primarily theoretical); (4) long higher education (15+ years) (primarily theoretical).

† With Bonferroni adjustment for multiple tests.

‡ Macronutrient energy percentages are calculated including alcohol.

§ Sugar content >10 g per 100 g.

║ Burger, toast, spring roll, hotdog, French hotdog, sandwich, filled croissant, pizza, falafel, humus, filled patty shell.

¶ Chips, popcorn, groundnuts, pistachio nuts, almonds, cheese dippers, pretzels.

** Sum of the variables snacks and sweets and chocolate as well as chips, confectionery, ice cream and desserts.

†† Sugar-sweetened beverages, cider, ice tea.

In Kruskal–Wallis analyses for differences between children in the lowest, medium and highest age-dependent tertiles of sleep duration, only intake of liquid ‘discretionary calories’ was significantly lower in long sleepers than in short and medium sleepers (*P =* 0·03), as seen in Table 3. However, there was a tendency towards a higher intake of dietary fibre (*P* = 0·09) and lower intake of added sugars (*P* = 0·10) in long sleepers (Table 4).

**Table 3.** Intake of food groups in 4- to 14-year-old children with short (*n* 268), medium (*n* 267) and long (*n* 267) sleep duration (Mean values and standard deviations)

Sleep duration…	Short		Medium		Long		
	Mean	sd	Mean	sd	Mean	sd	P*
High-fat milk and milk products (g/d)	16	62	23	77	25	104	0·59
Low-fat milk and milk products (g/d)	187	256	220	247	180	217	0·25
Rye bread (g/d)	49	36	49	34	53	37	0·29
Wheat bread (g/d)	72	42	72	43	71	42	0·87
Sugary breakfast cereals (g/d)†	2·5	6	3·3	6	3·0	7	0·14
Fruit (g/d)	181	138	177	124	178	138	0·82
Vegetables (g/d)	122	75	129	70	131	75	0·23
Meat and meat products (g/d)	90	42	92	39	88	41	0·34
Poultry (g/d)	19	19	18	18	18	20	0·42
Fish and seafood (g/d)	12	15	11	14	12	13	0·86
Fast food and light meals (g/d)‡	78	75	88	83	80	76	0·27
Snacks (g/d)§	8	12	8	15	7	11	0·86
Sweets and chocolate (g/d)	22	22	21	18	22	22	0·97
Solid ‘discretionary energy’ (g/d)∥	95	58	90	53	88	53	0·32
Liquid ‘discretionary energy’ (g/d)¶	244	219	242	219	214	224	0·03

* Kruskal–Wallis test for difference between age-dependent tertiles of sleep duration.

† Sugar content >10 g per 100 g.

‡ Burger, toast, spring roll, hotdog, French hotdog, sandwich, filled croissant, pizza, falafel, humus, filled patty shell.

§ Chips, popcorn, groundnuts, pistachio nuts, almonds, cheese dippers, pretzels.

║ Sum of the variables snacks and sweets and chocolate as well as chips, confectionery, ice cream and desserts.

¶ Sugar-sweetened beverages, cider, ice tea.

**Table 4.** Intake of nutrients in 4- to 14-year-old children with short (*n* 268), medium (*n* 267) and long (*n* 267) sleep duration (Mean values and standard deviations)

Sleep duration…	Short		Medium		Long		
	Mean	sd	Mean	sd	Mean	sd	P*
Energy (MJ/d)	8·3	2·3	8·3	2·0	8·2	2·2	0·69
Protein (g/d)	69	20	69	18	68	19	0·75
Fat (g/d)	76	26	74	21	74	25	0·84
SFA (g/d)	32	11	32	9·9	32	12	0·83
MUFA (g/d)	26	9·2	25	7·5	25	8·9	0·66
PUFA (g/d)	11	3·8	11	3·0	11	3·5	0·92
Carbohydrate (g/d)	266	76	264	67	262	72	0·68
Added sugars (g/d)	63	33	61	31	59	32	0·10
Dietary fibre (g/d)	15	2·7	17	5·0	18	6·1	0·09

* Kruskal–Wallis test for difference between age-dependent tertiles of sleep duration.

## Discussion

This is the first publication with representative population data on sleep duration and its relationship with dietary intake in Danish children. We found a negative association between sleep duration and BMI in children aged 4–14 years, which is in accordance with several other studies^(^^2^^–^^4^^,^^6^^)^.

There was no significant association between sleep duration and energy intake, and children with short, medium and long sleep duration did not differ with regard to energy intake. This observation is in agreement with what has been found previously in adults^(^^6^^)^, implying that a small chronic energy gap associated with short sleeping is difficult to capture with the dietary assessment methods that are normally used in epidemiological research. When assessing the relationship between sleep duration and dietary variables with multiple linear regression analyses with BMI, age, sex, energy intake and parental education as covariates, we only found tendencies that intakes of vegetables and dietary fibre were positively associated with sleep duration, and that intakes of poultry and liquid ‘discretionary calories’ were negatively associated with sleep duration. Furthermore, when assessing differences between children in the lowest, medium and highest age-dependent tertiles of sleep duration, only intake of liquid ‘discretionary calories’ was significantly lower in long sleepers than in short and medium sleepers. There was, though, a tendency towards a higher intake of dietary fibre and lower intake of added sugars in long sleepers.

Mutual adjustment for vegetables when assessing the association between intake of dietary fibre and sleep duration and adjustment of dietary fibre when assessing the association between intake of vegetables and sleep duration weakened both associations markedly (data not shown), suggesting that vegetables and dietary fibre are interdependently associated with sleep duration. It might be speculated that discretionary sugar and/or caffeine from liquid ‘discretionary calories’ may act as a stimulant keeping children longer awake, thereby contributing to explaining the inverse association between liquid ‘discretionary calories’ and sleep duration.

With increasing age, there is an increasing autonomy in the dietary intakes of children, and in the pubertal period, which is a period of high general autonomy, the propensity for buying energy-dense foods might be high. But in younger ages it can be speculated that less sleep and higher intake of energy-rich foods are reflecting an overall lifestyle and that healthy behaviours are less relevant in the family. Other factors could also be considered, such as family upbringing, personal values and attitudes towards healthy eating habits and sleep hygiene, late-night activities such as screen time, for example, television viewing and computer games, daytime napping, and physical activity. Furthermore, compensating for insufficient weekday sleep during the weekends may also in part ameliorate the risk of childhood overweight^(^^18^^)^. In the present study, sleep duration was based on the average sleep duration during the week, and differences on weekdays compared with weekend days were not taken into account. The variability in sleep patterns during the week and the potential effect of ‘catch-up’ sleep need to be further studied.

Only a few other studies have examined the relationship between sleep duration and dietary intakes^(^^4^^,^^13^^–^^15^^,^^19^^,^^20^^)^. The results from these studies are concordant with those found in the present study, suggesting that inadequate sleep may partly be associated with less healthy food habits in children. In 10- to 11-year-old Finnish children, inadequate sleep was associated with a greater likelihood of consuming energy-rich foods and less likelihood of consuming nutrient-dense foods^(^^15^^)^. Further, sleepiness during daytime was associated with daily snacking in Japanese school girls, and with skipping breakfast and evening snacks in boys^(^^19^^)^. In another study on Taiwanese adolescents, adequate sleep was associated with adopting a healthy diet, including eating breakfast daily, eating three meals per d, choosing foods with little oil, and drinking at least 1·5 litres of water per d^(^^20^^)^. In 14- to 18-year-old Americans, a positive association between daytime sleep, which may have reflected an increased need for nocturnal sleep, and greater food cravings was found^(^^14^^)^. Finally, among Iranian school girls, those who were overweight had shorter sleeping times and consumed ‘less nutritious food’, such as candies, chocolates and potato chips, more often than normal-weight girls^(^^13^^)^. Although based on differing populations with different age groups and dietary intake variables, the general findings from studies of sleep duration and diet indicate more unfavourable dietary patterns in short sleepers. However, in a study of German children and adolescents no association was observed between sleep duration and a nutrition quality score reflecting consumption of healthy and unhealthy foods^(^^4^^)^. Since the hedonic value of food intake might play a crucial role in the association between short sleep duration and overweight, this is an important target of future research^(^^11^^)^.

The observed inverse association between BMI and parental education is consistent with the results from other studies in children and adolescents^(^^21^^)^. However, the fact that parental education was not associated with sleep duration in the present study is inconsistent with the findings of others^(^^22^^)^, and might be explained by the fact that only 10 % of the participating children had parents with educational level 1 (the lowest level, basic school). This might be insufficient to detect a difference. Although sex differences have been observed in some other studies examining the association between diet and sleep^(^^4^^,^^14^^,^^15^^)^, there were no sex differences regarding sleep duration, and adjusting for sex in the multiple linear regression analyses did not change the results markedly.

Among the limitations of the present study is its cross-sectional nature that cannot disentangle cause and effect. To confirm a causal relationship between sleeping habits and eating patterns, and to determine whether short sleep duration leads to greater dietary intake or whether greater intake leads to shorter sleep duration, prospective, longitudinal studies are needed. Moreover, BMI was calculated from parent-reported height and weight, and might therefore be slightly biased. Few studies have investigated this bias and findings have been inconsistent^(^^23^^,^^24^^)^. However, several studies have found relatively good relationships between measured and self-reported estimates of children's and adolescents' height and weight^(^^25^^,^^26^^)^. Additionally, we have no data on pubertal status, which might also influence both BMI^(^^16^^)^ and sleep duration^(^^27^^)^ and maybe also eating habits as well. However, no association between sleep duration and pubertal status has been found in obese children^(^^28^^)^. Possibly, under-reporting may have blurred the results particularly in the oldest age group, as it has been well documented that under-reporting increases with the age of children^(^^29^^)^, and occurs especially with food items perceived as unhealthy^(^^30^^)^. Finally, data were collected over a period of 5 years, in which some variables in the environment might have changed, such as more electronic devices that might take time from sleep.

The strengths of the present study include its nationwide character, as it is based on a nationally representative population study. We also had access to very detailed dietary data that enabled us to analyse the association of sleep duration with specific dietary variables. An additional strength of the present study is that sleep duration was reported every day for seven consecutive days and reported as a mean of 7 d and not as a measure of usual sleep duration. However, sleep duration was self-reported and not measured, but self-reported sleep duration has been found to be positively correlated with polysomnographic measurements^(^^31^^)^. There may be a potential overestimation of sleep duration in our data because parental reports of sleep duration may be determined by the time of going to bed and getting up rather than on actual time of sleeping. However, sleep diaries have been found to have better agreement with objective measurements than sleep questionnaires^(^^32^^)^, and therefore the quality of the sleep data in the present study may be considered relatively high.

In conclusion, there was a negative association between sleep duration and BMI in the present sample of 4- to 14-year-old Danish children. However, sleep duration was not associated with energy intake and the proposal that children with short sleep duration have less healthy eating habits than children with longer sleep duration was only weakly supported by the present findings. Although causality needs to be elucidated further, the present findings are contributory to gaining a better understanding of the link between sleep duration, dietary intake and body weight, which may be valuable for further research and health strategies related to the prevention of overweight and obesity.
